# A novel antibiotic combination of linezolid and polymyxin B octapeptide PBOP against clinical *Pseudomonas aeruginosa* strains

**DOI:** 10.1186/s12941-022-00531-5

**Published:** 2022-08-29

**Authors:** Ting Huang, Mao Zeng, Huiyao Fu, Kelei Zhao, Tao Song, Yidong Guo, Jingyu Zhou, Longfei Zhai, Chaolan Liu, Balakrishnan Prithiviraj, Xinrong Wang, Yiwen Chu

**Affiliations:** 1 Antibiotics Research and Re-Evaluation Key Laboratory of Sichuan Province, School of Pharmacy, Chengdu University, Chengdu, 610052 Sichuan China; 2 Department of Chemistry and Biochemistry, University of California, San Diego, La Jolla, CA 92093 USA; 3 Marine Bio-Products Research Laboratory, Department of Plant, Food and Environmental Sciences, Dalhousie University, Truro, NS B2N 5E3 Canada

**Keywords:** Polymyxin B octapeptide, Linezolid, Antibacterial activity, Synergistic effect, *Pseudomonas aeruginosa*

## Abstract

**Background:**

Antibiotic-resistant Gram-negative bacteria are becoming a major public health threat such as the important opportunistic pathogen *Pseudomonas aeruginosa* (*P. aeruginosa*). The present study investigated enhancement of the linezolid spectrum, which is normally used to treat Gram-positive bacteria, at inhibiting *P. aeruginosa* growth.

**Methods:**

The checkerboard test or time-kill assay were carried out to determine the antibacterial effects of linezolid in cooperation with polymyxin B octapeptide PBOP (LP) against *P. aeruginosa* based on in vitro model. The protective effect of LP against *P. aeruginosa* infection was assessed based on a *Caenorhabditis elegans* (*C. elegans*) model.

**Results:**

The synergistic activity and antibacterial effects were significantly increased against *P. aeruginosa* by LP treatment, while linezolid and PBOP as monotherapies exhibited no remarkably bactericidal activity against the clinical strains. Additionally, LP treatment modified biofilm production, morphology, swimming motility of *P. aeruginosa*, and protected *C. elegans* from *P. aeruginosa* infection.

**Conclusions:**

This research demonstrates that LP combination has significant synergistic activity against *P. aeruginosa*, and PBOP is potential to be an activity enhancer. Notably, this strategy improved the antibacterial activity spectrum of linezolid and other anti-Gram-positive agents and represents an effective choice to surmount the antibiotic resistance of bacteria in the long term.

**Supplementary Information:**

The online version contains supplementary material available at 10.1186/s12941-022-00531-5.

## Background

*Pseudomonas aeruginosa* (*P. aeruginosa*) is a versatile Gram-negative pathogen that causes brutal respiratory and healthcare-associated infections, and it is an important pathogen for immunocompromised and cystic fibrosis patients [1]. This bacterium is exceedingly difficult to eliminate once it colonizes and infects in the respiratory tract [2]. Carbapenem is an effective treatment for serious hospital infections caused by *P. aeruginosa* and other Gram-negative pathogens, which are difficult to treat [3]. However, the substantial increase in the occurrence of carbapenem-resistant *P. aeruginosa* (CRPA) has caused a global epidemic in hospital, and led to high morbidity and mortality rates [4]. Thus, it is urgent to develop and design innovative antibacterial agents for the effective treatment of *P. aeruginosa* infection.

Polymyxins are one of the few drug classes that are reliably active against most Gram-negative pathogens in worldwide [5]. Polymyxins are belonging to polycationic amphipathic peptide antibiotics that were discovered in the 1940s and are primarily bactericidal to Gram-negative bacteria [6]. Polymyxins exert bactericidal activity via binding to the outer membrane to disorganize the structure of Gram-negative bacteria, which leading to leakage of cytoplasmic components and cell death [7]. Although polymyxins are effective activity against abundant Gram-negative pathogens, just two polymyxins (polymyxin B and polymyxin E) are accessible in clinical therapy and are considered as the last-line treatment [8]. The nephrotoxicity and neurotoxicity of polymyxins compounds are the common obstacles preventing the clinical application worldwide. Polymyxin B octapeptide (PBOP) was considered as an effectual permeabilizers of the outer membrane in various Gram-negative bacteria with low concentration [9]. To reduce toxicity and retain bactericidal activity, several studies showed that combinations of PBOP, polymyxin E with traditional antibiotics and the anti-Gram-positive bacterial agent vancomycin exhibited excellent synergistic activity against Gram-negative pathogens, such as CRPA [7, 10, 11]. These findings highlight the rational design combined of polymyxin derivatives with other anti-Gram-positive bacterial drugs for the treatment of PA infections. Linezolid is the first clinical agent from the synthetic oxazolidinone antibiotics [12]. This drug exhibits effective antimicrobial activity against a great majority of Gram-positive bacteria, such as enterococci, staphylococci, streptococci, and species resistant to conventional antibiotics [13]. Similar to other oxazolidinones, linezolid impedes the process of bacterial protein synthesis via binding to the 23S RNA peptidyl transferase of bacteria or binding to the peptidyl site on 70S initiation complexes [14, 15]. These actions ultimately produce bacteriostatic effects by preventing the translation of mRNA [16]. Notably, the cross-resistance among linezolid and some common protein synthesis inhibitors is not detected in bacteria resistant to existing antimicrobial agents [17]. In recent years, linezolid exhibits great synergistic and antibacterial activity against multidrug-resistant pathogen infections by combined with other antibiotics [18, 19]. For instance, the antibacterial efficacy of linezolid was significantly improved when combined with rifampicin, which support the potential of linezolid in the development of new combinations of therapeutic agents.

In this study, the antibacterial activity of linezolid monotherapy and LP combination (linezolid and PBOP) against *P. aeruginosa* were investigated by checkerboard test and time-kill assay. The protective effect of LP was assessed with *P. aeruginosa* infection based on a murine alveolar macrophage cell model and a *Caenorhabditis elegans* (*C. elegans*) model. Our study provides an innovative strategy to control *P. aeruginosa* infections and may facilitate the design and development of effective therapeutic agents against *P. aeruginosa*-caused diseases.

## Methods

### Chemicals and reagents

Polymyxin B and ficin enzyme were obtained from Sigma-Aldrich (St. Louis, MO, USA). Polymyxin E was obtained from Kangmanlin (Nanjing, China). The following antibiotics were purchased from Yuanye Bio-technology Co., Ltd (Shanghai, China): erythromycin, lincomycin, vancomycin, ampicillin, ofloxacin, or Meilun (Dalian, China): linezolid, nisin, penicillin G, ciprofloxacin, tigecycline, and chlortetracycline.

### Bacteria, cells, and the growth conditions

*P. aeruginosa* 14 (PA14) was preserved in the laboratory [2]. *P. aeruginosa* ATCC27853 strain was kindly supplied by Dr. Min Wu [20]. The multidrug-resistant clinical strains *P. aeruginosa* PA-COP2 (PA2) and carbapenem-resistant *P. aeruginosa* B41 (PAB41) were isolated from separate patients with chronic obstructive pulmonary disease (COPD) and preserved in the laboratory [21, 22]. These strains were grown in LB (Luria–Bertani) medium or LB agar or M8 supplemented medium at 37 °C [23]. The MH-S cell, a wide use cell line of murine alveolar macrophage, was cultured according to our previously report [24].

### *C. elegans* and growth condition

*C. elegans* strain N2 was stored in the current laboratory [21] and was maintained on nematode growth medium (NGM) by seeding with the *Escherichia coli* OP50 strain at 20 °C incubator.

### Preparation of polymyxin B and polymyxin E derivatives

The polymyxin B and polymyxin E derivatives were prepared according to the published reports [9, 25]. Briefly, two grams of polymyxin B or polymyxin E was dissolved in 150 mL of phosphate-buffered saline (PBS, 0.1 M, pH 7.0) and 50 mg of ficin (40 units·mg^−1^) was added. The mixture was treated at 37 °C incubator for 6 h with shaking at ~ 50 rpm and then was stirred in boiling water bath for 5 min, and the formed precipitate (denatured ficin) was removed. The solution was acidified with 50 μL of HCL (1 mol/L), washed twice with 75 mL of ethyl acetate, and then the water-phase solution was concentrated by rotary evaporator. The concentrated solution was loaded on a C-18 column, and products were eluted using acetonitrile: PBS at ratio of 15:85 (PBS, 0.1 M and containing 20 g/L sodium sulfate, pH 7.0). The purity of products was analyzed using high-performance liquid chromatography (HPLC). Afterwards, the structures of the polymyxin B and polymyxin E derivatives were identified by nuclear magnetic resonance spectroscopy (NMR) and mass spectrometry (MS) as described previously [26]. The polymyxin B and polymyxin E derivatives were shown as follows: polymyxin B heptapeptide (PBHP), PBOP, polymyxin B nonapeptide (PBNP), polymyxin E heptapeptide (PEHP), polymyxin E octapeptide (PEOP) and polymyxin E nonapeptide (PENP). The final purified products of polymyxin B and polymyxin E derivatives were concentrated and lyophilized.

### Susceptibility tests

The MICs (minimal inhibitory concentrations) were determined for different *P. aeruginosa* strains using micro-dilution method, with a concentration of 1 × 10^5^ CFU/mL on LB broth for 24 h, according to the published procedures [27]. The experiment was carried out in three repeats.

### Antibiotic synergy test

Synergistic effect of the 6 polymyxin B or polymyxin E derivatives in combination with 5 conventional antibacterial agents was detected using the checkerboard method as described on the previous study [28]. Briefly, the serial concentrations of test compounds were prepared by a two-dimensional array. The tested dilutions were based on the MICs of the two different compounds. The fractional inhibitory concentration index (FIC) was applied to illuminate the checkerboard test based on formulas as follows: FIC Index = FIC_A_ + FIC_B,_ FIC_A_ = MIC_A+B_/MIC_A_, and FIC_B_ = MIC_B+A_/MIC_B_. The value of MIC_A+B_ is indicated as the MIC value of Compound A with Compound B, and equally for MIC_B+A_. FIC index values were defined as synergy (FIC index ≤ 0.5), no obvious interaction (FIC index > 0.5–4.0), and antagonism (FIC index > 4.0). The experiments were repeated in triplicate.

### Biofilm production

The biofilm production was determined using a common crystal violet staining method and was quantified at OD_595_ value as described in the previous reports [29, 30]. Briefly, 1.0 × 10^6^ CFU of PA14 strain was incubated in a 24-well plate or the glass tubes with LB medium and treated with PBS (pH 7.4), linezolid (8 μg/mL), PBOP (16 μg/mL) or LP respectively, at 37 °C for 12 h. Unattached bacteria and culture supernatant were gently discarded, and the plates or tubes were carefully cleaned with PBS for 3 times. Biofilms were stained with crystal violet solution (0.2%, wt/vol)) for 1 h and were quantified at OD_595_ after dissolution with 95% ethanol. The experiments were repeated in triplicate.

### Scanning electron microscopy (SEM)

PA14 (1.0 × 10^6^ CFU) strain was cultured in a 24-well plates with LB medium and treated with PBS, linezolid (8 μg/mL), PBOP (16 μg/mL) or LP respectively, at 37 °C incubator for 12 h as mentioned above. The morphological characteristic was examined using scanning electron microscopy as previously reported [31].

### Bacterial motility assay

The swimming, swarming and twitching motilities of PA14 were determined by inoculating or stabbing bacterial cells on modified M8 or LB plates containing 0.3%, 0.5% or 1.0% agar powder and added PBS, linezolid (8 μg/mL), PBOP (16 μg/mL) or LP respectively, at 37 °C for at least 12 h, as previously described [23]. Briefly, for swimming motility, the PA14 inoculum was stabbed into the agar layer of a 0.3% M8 plate by a sterile toothpick. For swarming motility, the PA14 inoculum was spotted on the agar surface of a 0.5% M8 plate. For twitching motility, in the center of 1.0% LBA plate, the PA14 inoculum was stabbed perpendicularly to the agar-plastic interface at the bottom of the plate. The colony diameter of PA14 was measured using a ruler. All experiments were repeated in triplicate.

### Infection experiments in the MH-S cell model and *C. elegans* model

PA14 strain was cultured with LB medium at 37 °C incubator for 14 h to 16 h. The pellet of PA14 was collected via centrifugation (5000×*g*) and was resuspended in sterilized PBS. MH-S cells were maintained in RPMI-1640 medium supplemented with 10% fetal bovine serum at 37 °C incubator with 5% CO_2_. The infection experiments based on a MH-S cells model were carried out as previous reported [2]. In brief, the MH-S cells were grown in a medium without antibiotic and were infected with PA14 at an MOI of 10:1 (the ration of bacteria-cell) for 1 h. Afterwards, PBS, linezolid (8 μg/mL), PBOP (16 μg/mL) or LP were added into medium for 1 h, 6 h and 12 h, respectively. The cell viability of MH-S cells was detected using a Cell Counting Kit-8 (CCK8 kit) (Mei5 Biotechnology, Beijing, China) [32]. The bacterial loads in the MH-S cells were assessed at indicated time as previously reported [2]. Briefly, the infected cells were carefully washed in PBS for 3 times and lysed in a Triton X-100 solution (0.2%, sterile). The bacterial CFUs were then counted on LB agar plates.

To determine the protective effect of LP, all groups of *C. elegans* were infected with PA14 in slow killing (SK) agar plate and were treated with PBS, linezolid (8 μg/mL), PBOP (16 μg/mL) or LP. The mortality of the infected *C. elegans* was evaluated for 7 days. Nematodes that did not show an obvious response to touch were considered dead. The bacterial loads inside the *C. elegans* were counted at 12 h or 24 h post-infection as previously described [33, 34]. Briefly, the infected nematodes were mixed with silicon carbide particles and vortexed at maximum speed for one minute. The resulting suspension was serially diluted and plated on a selective agar plate to determine the bacterial CFU. This experiment was performed in triplicate.

### Statistical analysis

All data were analyzed using a GraphPad Prism 5.0 software, including one-way analysis of variance (ANOVA), Tukey–Kramer post hoc test, and a Mantel-Cox log rank test as previously described [35]. A value of p < 0.05 was considered as significant.

## Results

### Antibiotic sensitivity and resistance patterns of selected antibiotics

All tested *P. aeruginosa* strains were exceedingly resistant the antibiotics, which were normally applied to treat Gram-positive bacteria such as lincomycin and linezolid (Table 1). Meanwhile, all the strains were sensitive to the antibiotics against common Gram-negative bacteria with a low concentration such as ciprofloxacin, ofloxacin as shown in Table 1. Nevertheless, all the tested strains were unsusceptible the 6 polymyxin derivatives (PBHP, PBOP, PBNP, PEHP, PEOP, and PENP)) and all MIC values were over 256 µg/mL.

**Table 1:** MICs of 4 *P. aeruginosa* strains tested by 11 antimicrobial agents

Antimicrobial agents	MIC (μg/mL)			
	*P. aeruginosa* ATCC27853	*P. aeruginosa* PA14	*P. aeruginosa* PA2	*P. aeruginosa* PAB41
Erythromycin	> 256	> 256	> 256	> 256
Lincomycin	> 256	> 256	> 256	> 256
Linezolid	> 256	> 256	> 256	> 256
Nisin	> 256	> 256	> 256	> 256
Vancomycin	> 256	> 256	> 256	> 256
Penicillin G	8	8	4	4
Ampicillin	4	4	4	8
Ciprofloxacin	1	0.5	0.5	1
Ofloxacin	0.5	0.5	0.5	1
Tigecycline	4	2	4	2
Chlortetracycline	4	4	4	8

### The synergistic effect combined polymyxin derivatives and conventional antibiotics against *P. aeruginosa*

As shown in Table 2, the synergistic effects of 6 polymyxin derivatives in combination with 5 anti-Gram-positive agents were detected for *P. aeruginosa* by a checkerboard assay. FIC Index values were analyzed by considering the absence of visible growth in all combinations. The lowest FIC value was presented in each combination group (Table 2 and Additional file 1: Table S1), and the values were in a wide ranged from 0.078 to 0.375. The synergistic effect was detected (FIC Index ≤ 0.5), while the antagonism was not observed in the combination groups (Table 2). Since the LP combination (linezolid and PBOP) had the lowest FIC value in all the tested group (Additional file 1: Table S1), we concentrated on LP as a new combination and determined their antibacterial activity in this work.

**Table 2:** FICs of 6 polymyxin derivatives combined with 5 antibiotics against *P. aeruginosa* PA14

Antimicrobial agents	FIC*				
	Erythromycin	Lincomycin	Linezolid	Nisin	Vancomycin
PBHP	0.281	0.188	0.141	0.375	0.141
PBOP	0.156	0.094	0.078	0.313	0.156
PBNP	0.188	0.156	0.156	0.250	0.250
PEHP	0.258	0.250	0.141	0.266	0.156
PEOP	0.313	0.141	0.188	0.313	0.125
PENP	0.281	0.125	0.281	0.375	0.188

^*^Combination of polymyxin derivatives and antibiotics giving the lowest FIC Index value

### LP reduced bacterial burdens in vitro

**Fig. 1:**
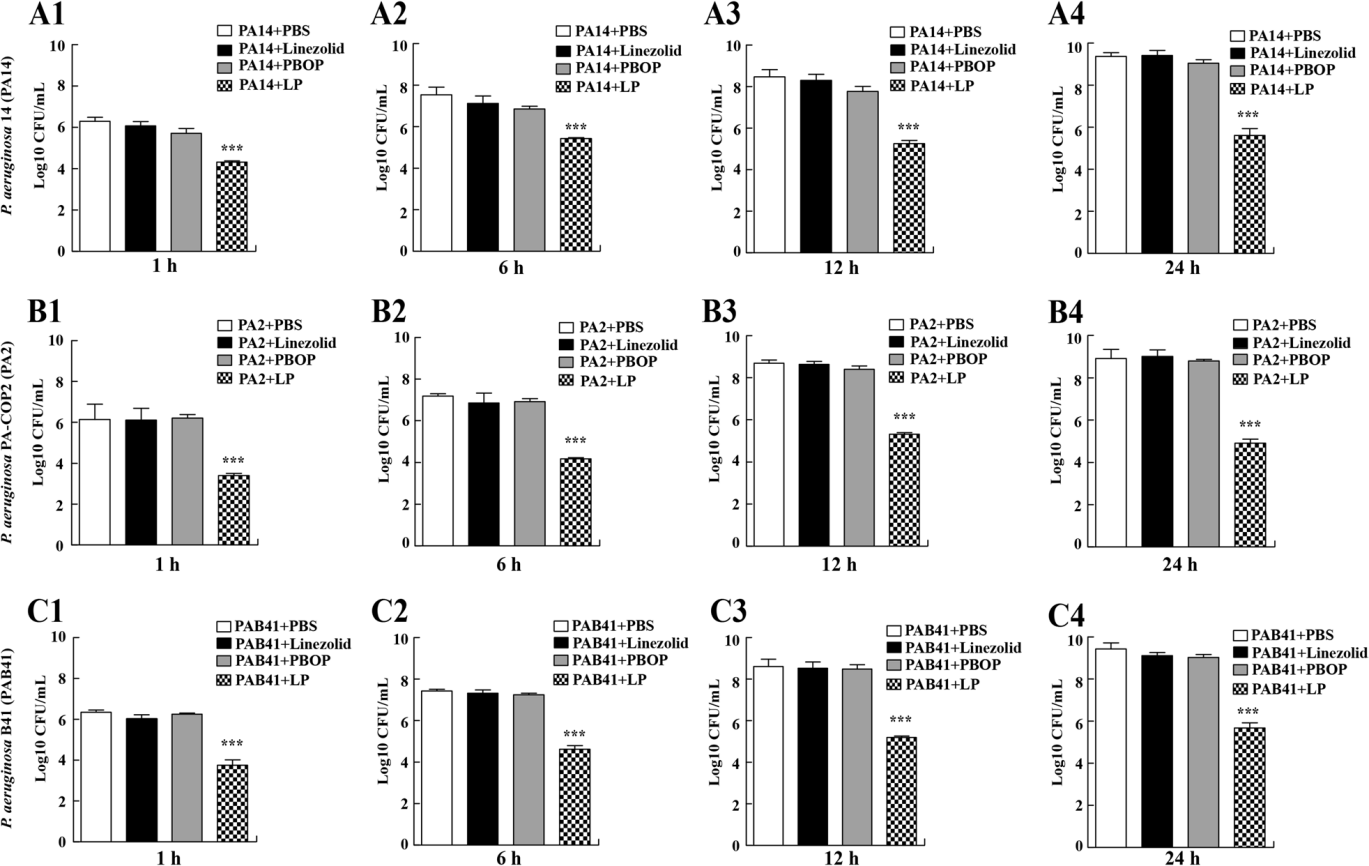
Treatment of LP reduced bacterial burdens. **A1**–**A4** 1 × 10^5^ CFU of PA14 were seeded in 96-well plates and treated with PBS, linezolid, PBOP or combination of linezolid and PBOP (LP) for 1 h (**A1**), 6 h (**A2**), 12 h (**A3**), and 24 h (**A4**). The bacteria burdens on the LB agar plates were counted. **B1**–**B4** 1 × 10^5^ CFU of PA2 were seeded in 96-well plates and treated with PBS, linezolid, PBOP or LP for 1 h (**B1**), 6 h (**B2**), 12 h (**B3**), and 24 h (**B4**). **C1**–**C4** 1 × 10^5^ CFU of PAB41 were seeded in 96-well plates and treated with PBS, linezolid, PBOP or LP for 1 h (**C1**), 6 h (**C2**), 12 h (**C3**), and 24 h (**C4**). The bacteria burdens were determined as described above. Data are shown as the mean ± SEM of three independent experiments. ***p < 0.001, **p < 0.01

To explore the antibacterial activity of LP combination in the *P. aeruginosa *in vitro, PA14 were cultured in 96-well plates and were treated with PBS, linezolid, PBOP or LP for 1 h, 6 h, 12 h, and 24 h. Our data indicated that bacterial CFU was declined in the LP treated group compared with control group at 1 h, 6 h, 12 h, and 24 h post exposure (Fig. 1, A1–A4). To validate these interesting data, we also detected the antimicrobial effects of LP on a multidrug-resistant clinical strain PA2 (Fig. 1, B1–B4) or a CRPA strain PAB41 (Fig. 1, C1–C4. Treatment with LP remarkably decreased the bacterial CFU at the indicated time points compared to the linezolid single-treated group and PBOP single-treated group (Fig. 1, B1–C4).

**Fig. 2:**
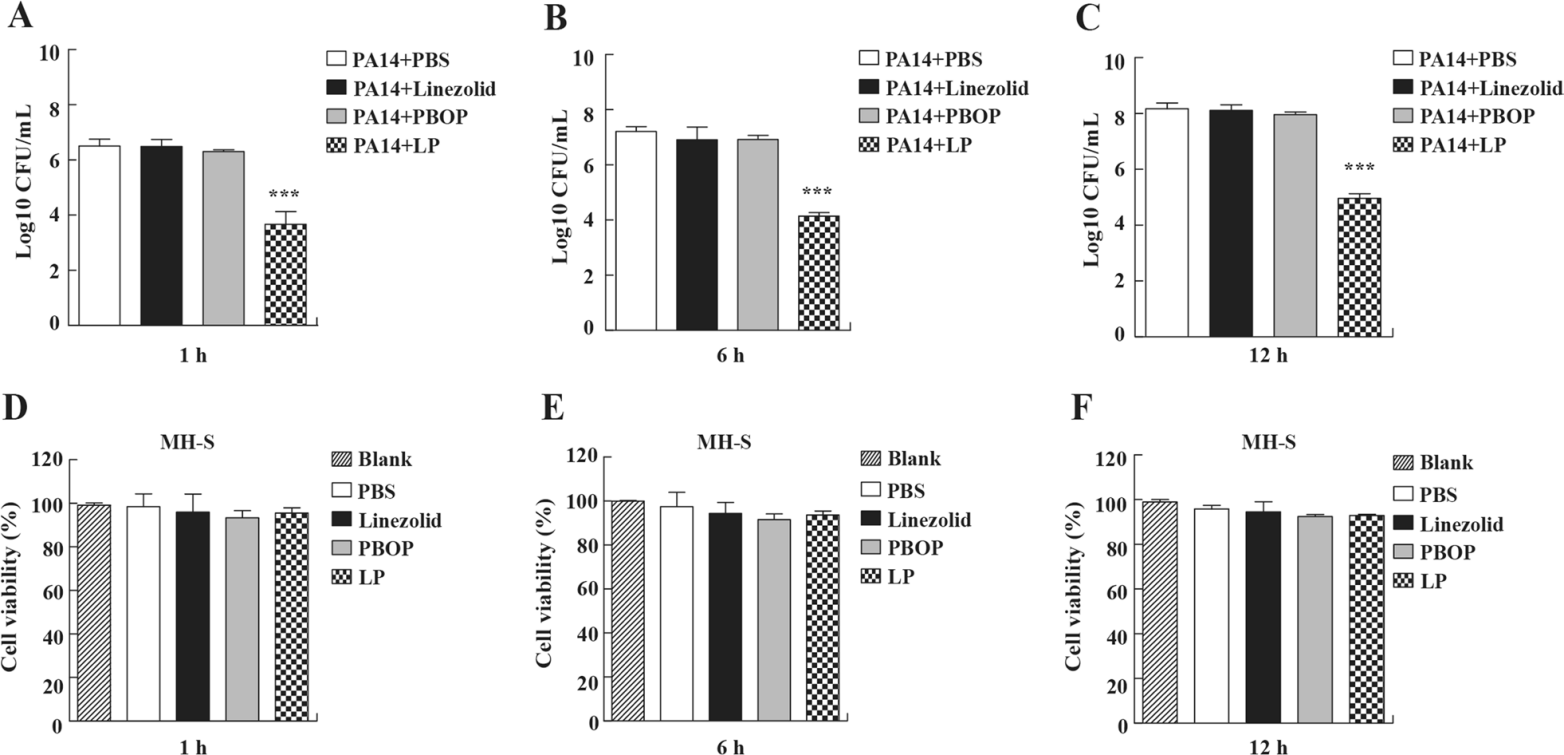
Bacterial burdens and cell viability in MH-S cell infected with *P. aeruginosa*. **A**–**C** The MH-S cells were infected with PA14 at an MOI of 10 for 1 h. And then treated with PBS, linezolid, PBOP or LP for 1 h (**A**), 6 h (**B**), and 12 h (**C**). The bacteria burdens were determined as described above. **D**–**F** MH-S cells were seeded in 96-well plates and treated with PBS, linezolid, PBOP or LP for 1 h (**D**), 6 h (**E**), and 12 h (**F**). The cell viability of MH-S cells was determined by CCK8 kit. Blank, the MH-S cells were not treated. Data are shown as the mean ± SEM of three independent experiments. ***p < 0.001, **p < 0.01

Macrophages are the predominant cells for making response to various bacterial infections. To determine the protective role of LP against PA14 infection, we examined its effects based on a MH-S cell model by counting the clearance of PA14. Our results demonstrated that LP treatment considerably promoted clearance of PA14 in MH-S cells at different infection time (Fig. 2A–C). In addition, we found that statistically significant differences were not detected in all the treated groups compared to blank group by the cell viability experiment (Fig. 2D–F), suggesting that LP have no prominent toxicity in cells.

### LP modified the biofilm production and morphology of *P. aeruginosa*

**Fig. 3:**
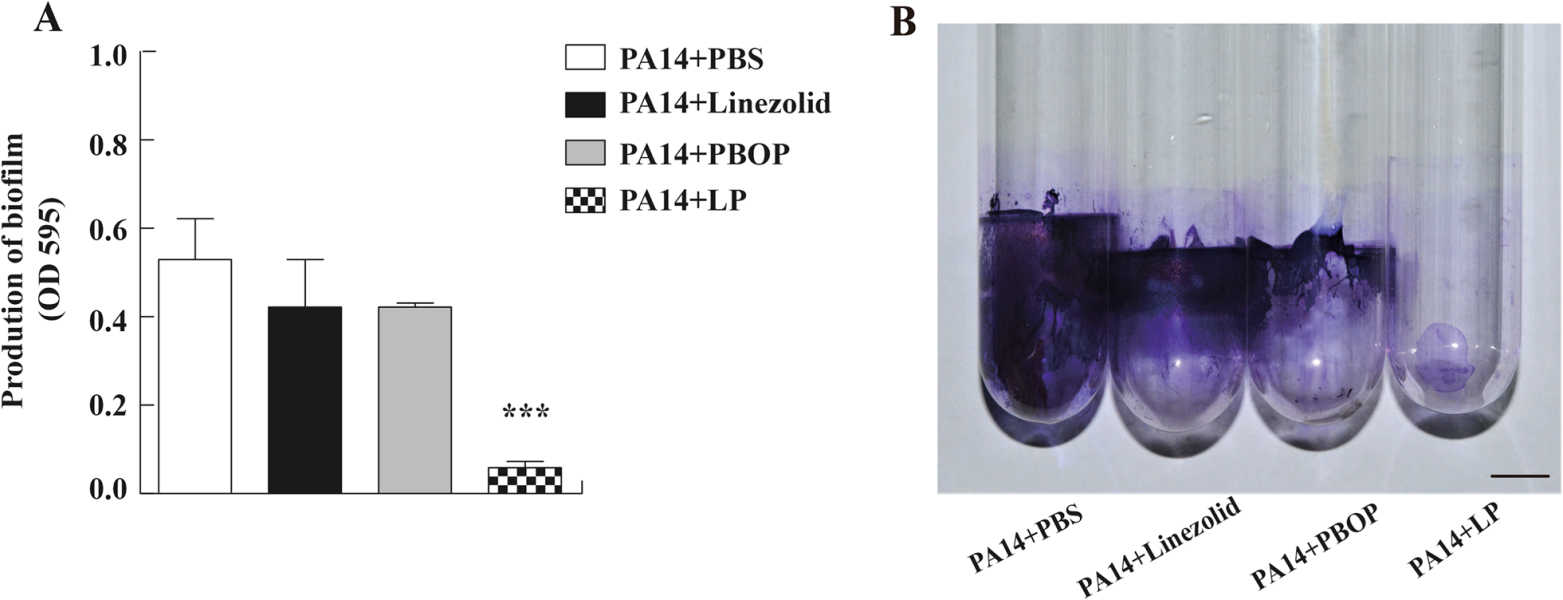
The biofilm production of *P. aeruginosa* treated by LP. **A**, **B** 1 × 10^6^ CFU of PA14 or were seeded in 96-well plates (**A**) or glass tubes (**B**) and treated with PBS, linezolid, PBOP or LP for 12 h. The biofilm production was quantified at OD_595_. Scale bar, 1 cm. Data are shown as the mean ± SEM of three independent experiments. ***p < 0.001

**Fig. 4:**
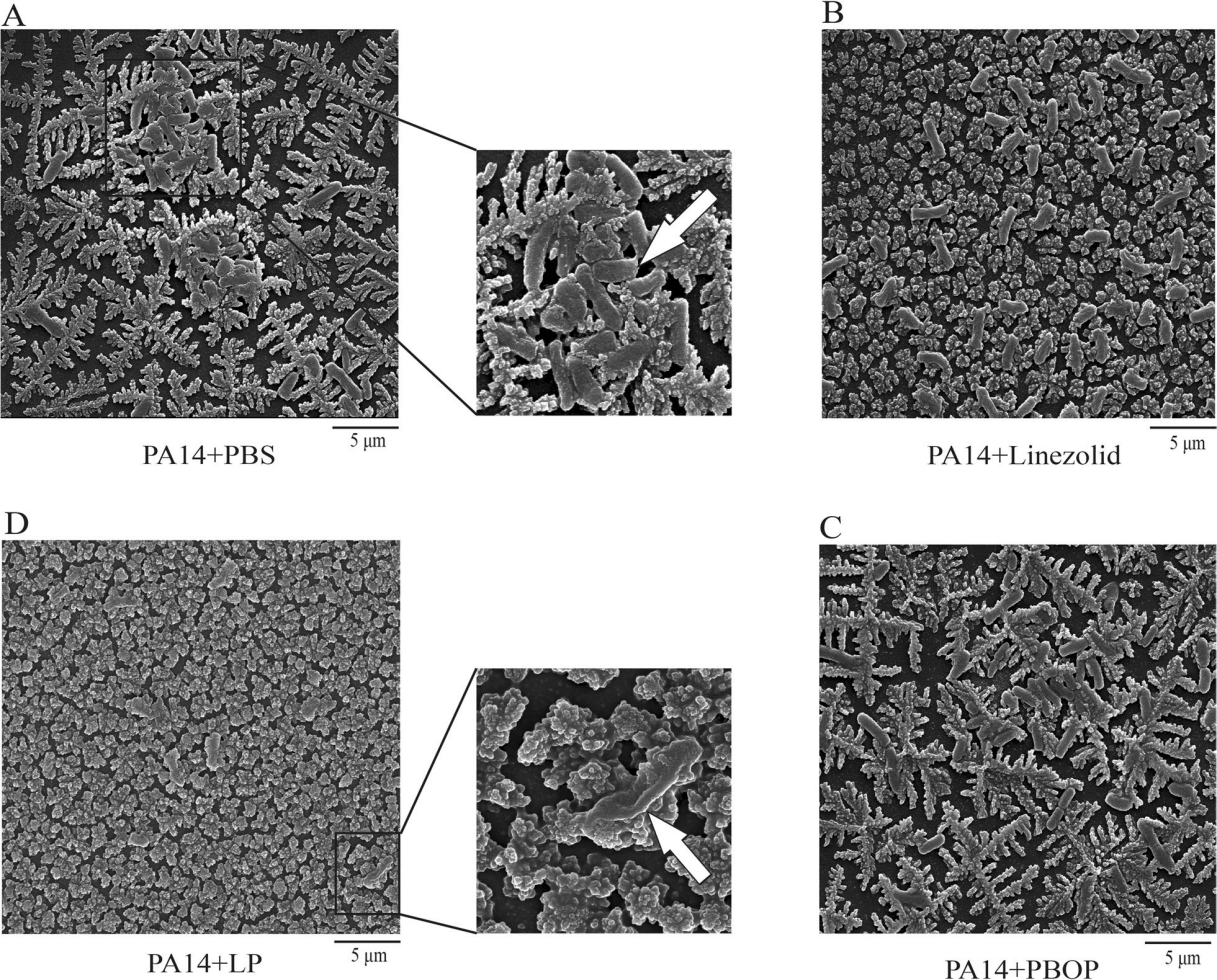
The morphology of *P. aeruginosa* treated by LP. **A**–**D** 1 × 10^5^ CFU of PA14 were seeded in 96-well plates and treated with PBS (**A**), linezolid (**B**), PBOP (**C**) or LP (**D**) for 12 h. The morphology of PA14 was detected by scanning electron microscopy (SEM). The destroyed PA14 is indicated by the arrow (D). Scale bar, 5 μm. All the experiments were performed in triplicate

Bacteria predominantly form biofilms, and it is hard to clean the biofilms with present antimicrobial agents. To investigate whether the biofilm production of *P. aeruginosa* was altered by LP treatment, PA14 strain was cultured in 96-well plates or glass tubes and were added with PBS, linezolid, PBOP or LP for 12 h. We found that LP treatment remarkably reduced the biofilm production of PA14 as shown in Fig. 3A, B. Additionally, to further investigate whether the morphology of *P. aeruginosa* was affected by LP, PA14 were cultured in a 96-well plates and were treated with PBS, linezolid, PBOP or LP for 12 h. As shown in Fig. 4D, the LP drastically altered the morphology of PA14 including cell lysis and cell shrinkage detected by SEM, however the morphology of PA14 in other groups wasn’t significantly changed (Fig. 4A–C).

### LP affected swimming motility of *P. aeruginosa*

**Fig. 5:**
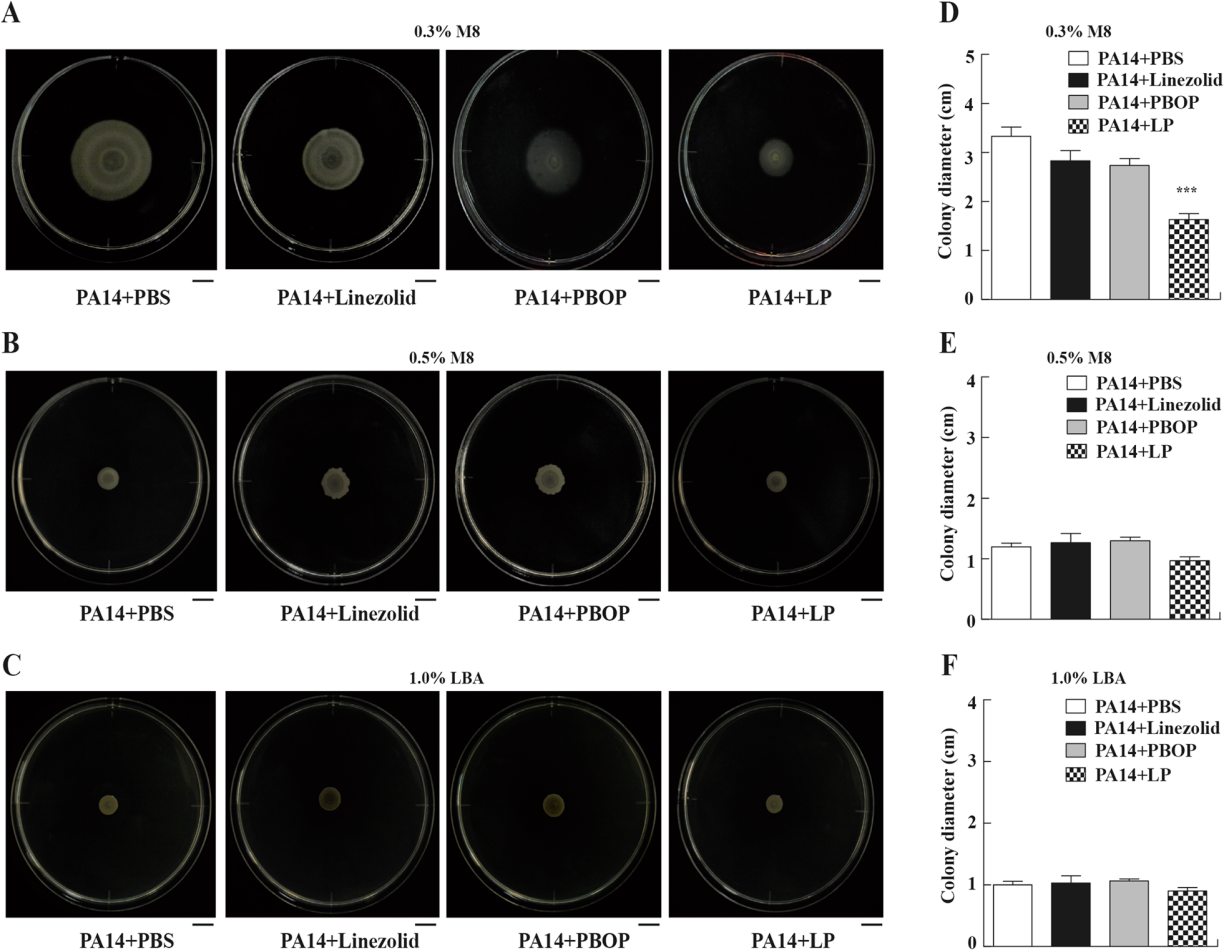
The motility of *P. aeruginosa* treated by LP. **A**–**F** 5 × 10^6^ CFU of PA14 were seeded in the center of 0.3% M8 plates (**A**, **D**), 0.5% M8 plates (**B**, **E**) or 1.0% LBA plates (**C**, **F**) and treated with PBS, linezolid, PBOP or LP for 24 h. The colony diameter of *P. aeruginosa* was measured by ruler (**D**–**F**). Scale bar, 1 cm. Data are shown as the mean ± SEM of three independent experiments. ***p < 0.001

Next, to study whether LP could affect the bacterial motility of *P. aeruginosa*, PA14 strain were seeded in 0.3% M8 plates, 0.5% M8 plates or 1.0% LBA plates and were added PBS, linezolid, PBOP or LP for 24 h. As shown in Fig. 5A, the LP remarkably inhibited swimming motility of PA14 in the 0.3% M8 plates. In addition, the colony diameter of PA14 treated by LP was significantly reduced in the 0.3% M8 plates compared to PBS-treated groups (Fig. 5D). However, the LP treatment might not affect the swarming motility or twitching motility of PA14 compared to PBS-treated groups in the 0.5% M8 plates (Fig. 5B, E) or the 1.0% LBA plates (Fig. 5C, F).

### LP promoted survival rate of *C. elegans* from *P. aeruginosa* challenge

**Fig. 6:**
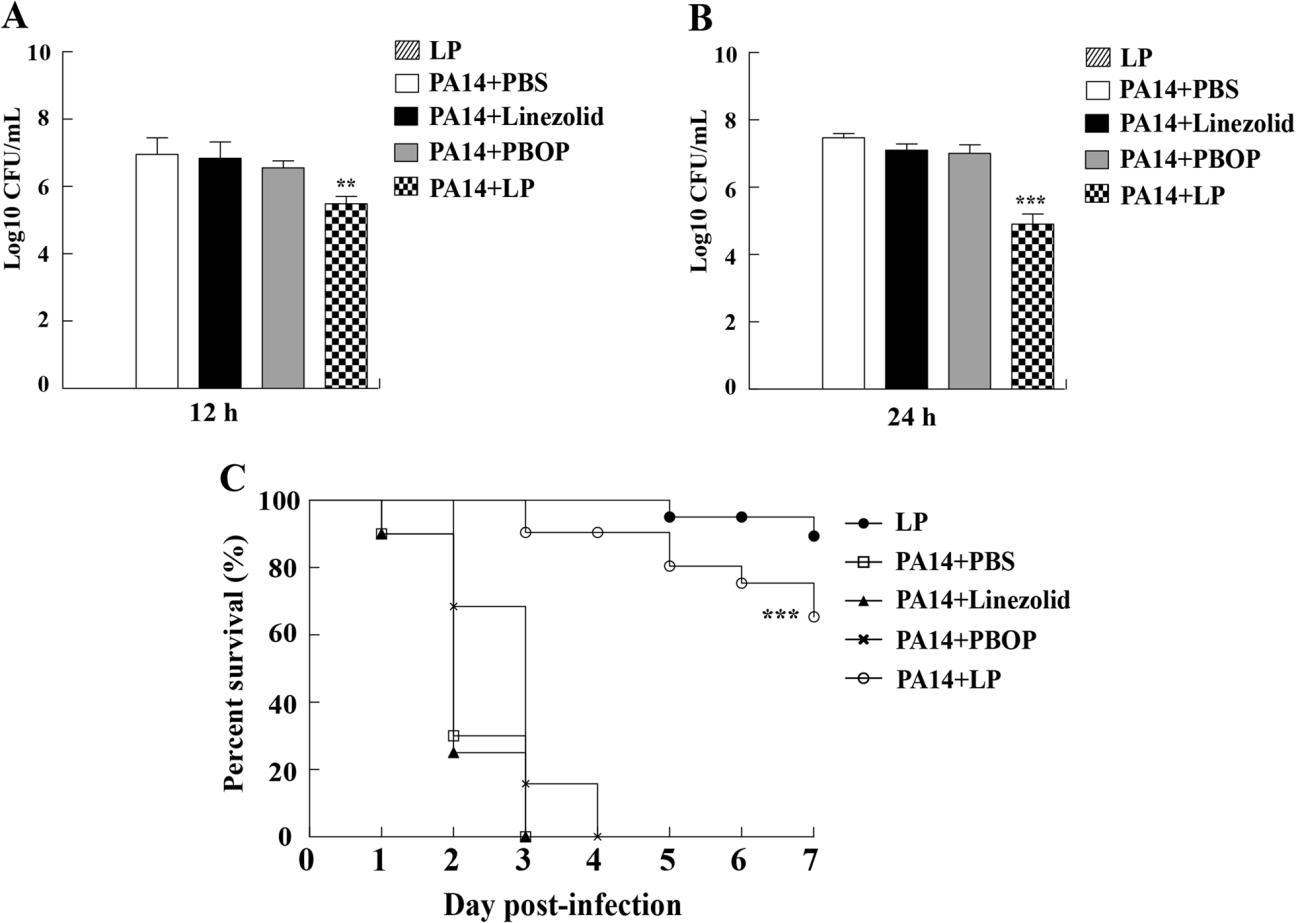
Residual CFUs and survival rate of *C. elegans* infected with *P. aeruginosa*. The *C. elegans* were infected at day 0 with PA14 and treated with PBS, linezolid, PBOP or LP for determining bacterial burdens at post treatment 12 h (**A**) and 24 h (**B**). The survival rate of *C. elegans* was monitored for the subsequent 7 days (**C**). PBS was used as a control. **p < 0.01 and ***p < 0.001

The *C. elegans* nematode is an effective model for high efficiency screening of antibacterial agents in *P. aeruginosa*. To determine the antibacterial effect of LP, *C. elegans* were infected with PA14 in SK plate and were treated with different drugs. The results showed that a great number of bacteria were detected in the PBS, linezolid or PBOP treated group (Fig. 6A, B). However, the *C. elegans* treated with LP exhibited a small number of bacteria in the *C. elegans* at 12 h (Fig. 6A) and 24 h (Fig. 6B) post infection. For the survival rates, the *C. elegans* from PBS, linezolid or PBOP monotherapy group died at day 3 as shown in Fig. 6C. Notably, lots of *C. elegans* survived the *P. aeruginosa* infection at days 7 post infection in the LP-treated group. Moreover, most uninfected *C. elegans* from LP group survived, suggesting LP did not have significant effect on the growth of *C. elegans* and LP might be not toxic in *C. elegans*.

## Discussion

Multidrug-resistant (MDR) Gram-negative bacteria such as MDR-PA poses a great challenge to public health care worldwide, and development of new drugs is urgent to against these bacteria in the present antibiotic pipeline [36]. For instance, treating infections caused by MDR-PA is becoming more and more difficult, since this versatile pathogen exhibits acquired or adaptive resistance to most commercial antibiotics [10]. Notably, the occurrence of MDR-PA is becoming increasingly predominant in low-income countries and only a few available therapeutic options remain [10]. Antibiotic combination therapy is widely investigated for some serious infections caused by MDR-Gram-negative bacteria, but useful data on the most effective combinations are limited. The current study, we successfully prepared a polymyxin B octapeptide (PBOP) and found that the combination of linezolid and PBOP exhibited a significant synergistic effect against Gram-negative bacterium *P. aeruginosa* and MDR-PA. Interestingly, the LP combination treatments greatly improved the antibacterial activities in vitro and showed the excellent protective effects against *P. aeruginosa* infections challenge based on a *C. elegans* model. Therefore, this combination therapy provides an innovative treatment strategy, and improves the spectrum of antibiotics and treatment outcomes.

The membrane-disrupting ability of polymyxins enables the entry of a second antibacterial agent, which increases the permeability and antibacterial activity. These effects are critical mechanism of the synergistic effects for the antibiotic combinations [37]. To the best of our knowledge, few published investigations focused on the synergistic and antibacterial activity of LP to combat *P. aeruginosa*. Although a recent study evaluated the antibacterial efficacy of linezolid in combination with polymyxin B against clinical MDR-PA in vitro, the potential harm or toxicity of polymyxin B in vivo may remain [37]. To alleviate the potential toxicity of polymyxins and retain their strong activity for membrane-permeabilizing, we prepared different derivatives of polymyxins using the proteolytic enzyme ficin. We tested several traditional anti-Gram-positive agents and various derivatives of polymyxins to combat *P. aeruginosa* and clinical MDR-PA by examined the synergistic and antibacterial activities of novel antibiotic combinations. We found that the *P. aeruginosa* strains were highly resistant to the selective anti-Gram-positive agents and different derivatives of polymyxins. However, all the tested *P. aeruginosa* strains were highly sensitive to the LP combination in comparison to other groups in the checkerboard assay, which indicated this drug combination may be a potential alternative for controlling and treating *P. aeruginosa* infections. Because the LP combinations exhibited the lowest FIC value in all of the treated groups, we further explored their antibacterial activity throughout the entire study.

The time-kill assay showed significant differences between single agents and LP combination treatments against PA14 or clinical MDR-PA. Even though the bacteriostatic activity of PBOP was not persisted, we found that linezolid in combination with PBOP exhibited maximal bactericidal activity. Importantly, the viability of MH-S cells in all of the treated groups was no significant different from the blank group, which suggested that LP had no prominent toxicity, and PBOP was safe for cells. These interesting results were similar to a published study, in which MDR-PA strains were treated with a combined therapy based on polymyxins [38]. Additionally, a previous research demonstrated that polymyxin E monotherapy did not completely clean the bacteria, while the combination of a synergistic antimicrobial significantly improved the killing of bacteria [39]. Linezolid is a powerful antimicrobial compound with effective activity against many Gram-positive bacteria. Nevertheless, a recent study showed the antibacterial activities of linezolid to combat Gram-negative bacteria after capping with a silica xerogel in a novel nanoformulation [40]. *P. aeruginosa* predominantly forms biofilms on surfaces or air–liquid interfaces, which may contribute to their colonization and resistance to antibiotics [36]. Moreover, swimming motility is contributed to the pathogenesis of *P. aeruginosa* infections [41]. Intriguingly, LP treatment greatly improved the antibacterial activity, and altered the biofilm production, morphology or swimming motility of *P. aeruginosa*, which may be effective in preventing the colonization and dispersal of *P. aeruginosa*. These interesting findings reveal that PBOP is a promising nominee as an antibiotic potentiator, and LP can be an excellent antibacterial combination for treating *P. aeruginosa*.

To further explore the underlying protection of LP against the challenge of *P. aeruginosa*, the *C. elegans* infection model was used as target. Our results demonstrated that the LP combination therapy exhibited better antibacterial activity against *P. aeruginosa* compared to other groups. Linezolid and PBOP were worked synergistically, and protective effects were greatly improved as shown in the third day of treatment. In addition, for the bacterial loads, a great number of *P. aeruginosa* were recovered from the *C. elegans* in the control group and monotherapy group, which indicated that either linezolid or PBOP monotherapy did not inhibit the growth of bacteria and exert the bactericidal activity. In contrast to the findings mentioned above, few bacteria were detected in the *C. elegans* by LP treatment, which suggests that most of the bacteria were cleared and finally raised the survival rates of the *C. elegans*. These findings verified the antibacterial activity of the LP combination detected in vitro assays and indicated that linezolid was a favorable component for the treatment of *P. aeruginosa* infections when it was incorporated with PBOP. A published research emphasized that linezolid had strong activity for treatment of Gram-negative anaerobic bacteria [42], while the present study implied that linezolid also had strong antibacterial activity against Gram-negative aerobic bacteria when used in combination with PBOP, which brought the interest in linezolid against other MDR Gram-negative bacteria. We conclude that PBOP functions as a potentiator in conbimation with linezolid to enhance antibacterial activity and protective effect against *P. aeruginosa* infections.

## Conclusions

The LP combination exhibited remarkably synergistic and antibacterial activities against *P. aeruginosa* based on different models in this study. The presence of PBOP enhanced the bactericidal effects of linezolid, implying PBOP may broaden the therapeutic range of linezolid. More importantly, combination of well-known antibacterial agents in a new design will provide a hopeful method to impede the increasing bacterial resistance to the commercial and conventional antimicrobial. Although the number of tested strains is a constraint of this work, further wide-ranging study in rodents by estimating the clinical application significance of the findings may contribute to control *P. aeruginosa* infection or related diseases.

## Supplementary Information

**Additional file 1: Table S1.** FICs of 6 polymyxin derivatives combined with 5 antibiotics against *P. aeruginosa*.

## Data Availability

The datasets used during the current study are available from the corresponding author on reasonable request.
