# Supplementary material for: A novel antibiotic combination of linezolid and polymyxin B octapeptide PBOP against clinical Pseudomonas aeruginosa strains
Source: Ann Clin Microbiol Antimicrob. 2022 Aug 29;21:38. doi: 10.1186/s12941-022-00531-5 (PMC9422153; doi:10.1186/s12941-022-00531-5)
Supplement: Supplementary file 1 — Additional file 1: Table S1. FICs of 6 polymyxin derivatives combined with 5 antibiotics against P. aeruginosa. [file 12941_2022_531_MOESM1_ESM.docx]

Table S1.　FICs of 6 polymyxin derivatives combined with 5 antibiotics against *P. aeruginosa*.

| Antimicrobial | MIC (μg/mL) | | FIC | | Antimicrobial | MIC (μg/mL) | | FIC | Antimicrobial | MIC (μg/mL) | | FIC | Antimicrobial | MIC (μg/mL) | | FIC | Antimicrobial | MIC (μg/mL) | | FIC |
| --- | --- | --- | --- | --- | --- | --- | --- | --- | --- | --- | --- | --- | --- | --- | --- | --- | --- | --- | --- | --- |
| Agents | Single-use | Best combination* | |  | Agents | Single-use | Best combination* |  | Agents | Single-use | Best combination* |  | Agents | Single-use | Best combination* |  | Agents | Single-use | Best combination* |  |
| PBHP | 256 | 64 | | 0.281 | PBHP | 256 | 32 | 0.188 | PBHP | 256 | 4 | 0.141 | PBHP | 256 | 64 | 0.375 | PBHP | 256 | 4 | 0.141 |
| Erythromycin | 512 | 16 | |  | Lincomycin | 512 | 32 |  | Linezolid | 512 | 64 |  | Nisin | 512 | 64 |  | Vancomycin | 512 | 64 |  |
| PBOP | 256 | 32 | | 0.156 | PBOP | 256 | 16 | 0.094 | PBOP | 256 | 16 | 0.078 | PBOP | 256 | 64 | 0.313 | PBOP | 256 | 32 | 0.156 |
| Erythromycin | 512 | 16 | |  | Lincomycin | 512 | 16 |  | Linezolid | 512 | 8 |  | Nisin | 512 | 32 |  | Vancomycin | 512 | 16 |  |
| PBNP | 256 | 32 | | 0.188 | PBNP | 256 | 32 | 0.156 | PBNP | 256 | 32 | 0.156 | PBNP | 256 | 32 | 0.250 | PBNP | 256 | 32 | 0.250 |
| Erythromycin | 512 | 32 | |  | Lincomycin | 512 | 16 |  | Linezolid | 512 | 16 |  | Nisin | 512 | 64 |  | Vancomycin | 512 | 64 |  |
| PEHP | 256 | 64 | | 0.258 | PEHP | 256 | 32 | 0.250 | PEHP | 256 | 32 | 0.141 | PEHP | 256 | 64 | 0.266 | PEHP | 256 | 32 | 0.156 |
| Erythromycin | 512 | 4 | |  | Lincomycin | 512 | 64 |  | Linezolid | 512 | 8 |  | Nisin | 512 | 8 |  | Vancomycin | 512 | 16 |  |
| PEOP | 256 | 64 | | 0.313 | PEOP | 256 | 32 | 0.141 | PEOP | 256 | 32 | 0.188 | PEOP | 256 | 16 | 0.313 | PEOP | 256 | 16 | 0.125 |
| Erythromycin | 512 | 32 | |  | Lincomycin | 512 | 8 |  | Linezolid | 512 | 32 |  | Nisin | 512 | 128 |  | Vancomycin | 512 | 32 |  |
| PENP | 256 | 64 | | 0.281 | PENP | 256 | 16 | 0.125 | PENP | 256 | 64 | 0.281 | PENP | 256 | 64 | 0.375 | PENP | 256 | 32 | 0.188 |
| Erythromycin | 512 | 16 | |  | Lincomycin | 512 | 32 |  | Linezolid | 512 | 16 |  | Nisin | 512 | 64 |  | Vancomycin | 512 | 32 |  |

*Combination of polymyxin derivatives and antibiotics giving the lowest FIC Index value.
